# Supplementary material for: WordCommentsAnalyzer: A windows software tool for qualitative research
Source: F1000Res. 2018 Sep 4;7:536. Originally published 2018 May 3. [Version 2] doi: 10.12688/f1000research.14819.2 (PMC6137411; doi:10.12688/f1000research.14819.2)
Supplement: Supplementary file 3 [file f1000research-7-17614-s0002.tgz › b2a79496-4ada-47c4-9682-a99ce867836d.zip!/Eight Iranian Health Journals/Journal of Health System Research _1.docx]

© Journal of Health System Research . This work is licensed under CC BY-NC 4.0 (https://creativecommons.org/licenses/by-nc/4.0/).

Volume & Issue: دوره 14، شماره 1: 1397

Title: بررسي وضعیت آلودگي باکتریایی دكمه‌هاي آسانسور و کامپیوترهای کتابخانه بیمارستان دکتر محمد کرمانشاهی در سال 1394

http://hsr.mui.ac.ir/index.php/jhsr/article/view/3083

مقدمه: بر اساس مطالعات اپیدمیولوژیک، سطوح محیطی مانند سطح تجهیزات بیمارستانی در انتقال میکروارگانیسم‌ها دخیل می‌باشد. این موضوع تأثیر مثبتی در افزایش تعداد موارد عفونت‌های بیمارستانی دارد. هدف از انجام پژوهش حاضر، بررسي ‎‎وضعیت آلودگي ‎‎ميكروبي ‎‎دكمه‌هاي ‎‎آسانسور و کامپیوترهای کتابخانه بیمارستان دکتر ‏محمد کرمانشاهی بود. روش‌ها: در این تحقیق توصیفی– مقطعی، 71 نمونه از دکمه‌های آسانسور، صفحه کلید، ماوس و دکمه پاور کامپیوترهای کتابخانه بیمارستان دکتر محمد کرمانشاهی گرفته شد و به آزمایشگاه انتقال یافت. سپس با استفاده از روش‌های تشخیصی استاندارد باکتری‌شناسی شامل رنگ‌آمیزی، تست‌های بیوشیمیایی و کشت در محیط کشت‌های افتراقی، انتخابی و اختصاصی، باکتری‌های گرم مثبت و منفی جداسازی گردید. یافته‌ها: آلودگی باکتریایی در همه نمونه‌های مورد بررسی مشاهده شد. در نمونه دکمه‌های آسانسور، بیشترین فراوانی به باسیل‌های گرم مثبت اسپوردار (باسیلوس) (21/48 درصد) اختصاص داشت و کلبسیلا پنومونیه (57/3 درصد) کمترین فراوانی را به خود اختصاص داد. 66/66 درصد از نمونه‌های کامپیوترها به استافیلوکوکوس کواگولاز منفی آلوده بودند و از 66/6 درصد آن‌ها استافیلوکوکوس کواگولاز مثبت (اورئوس) و میکروکوکوس جدا شد. نتیجه‌گیری: با توجه به جداسازی باکتری‌های پاتوژن و غیر پاتوژن از هر دو گروه نمونه‌ها، لزوم توجه بیشتر به رعایت اصول بهداشت فردی و اصول ضد عفونی صحیح و منظم سطوح وسایل و تجهیزات بیمارستان جهت کاهش آلودگی باکتریایی و بروز عفونت‌های بیمارستانی، ضروری به نظر می‌رسد.

Title: بررسی اثربخشی فرایند پخت بر اکراتوکسین A در برنج‌های رایج مصرفی

http://hsr.mui.ac.ir/index.php/jhsr/article/view/3105

مقدمه: اکراتوکسین A یکی از متابولیت‌های ثانویه قارچی است که امکان انتقال آن از مواد غذایی مانند برنج و فرآورده‌های مشتق شده از آن به جیره‌ غذایی انسان وجود دارد. هدف از انجام مطالعه حاضر، بررسی میزان اکراتوکسین A در نمونه برنج‌های رایج مصرفی جمع‌آوری ‌شده از دو شهر اصفهان و شیراز و تأثیر فرایند پخت به دو روش آبکش و کته در کاهش میزان اکراتوکسین در نمونه‌های دارای بالاترین مقدار آلودگی بود. روش‌ها: در این پژوهش، 30 نمونه از شش برند پرمصرف برنج شامل سه نمونه برنج داخلی و سه نمونه برنج خارجی به روش تصادفی ساده انتخاب شد و تحت دو روش پخت آبکش و کته در دمای جوش آب قرار گرفت. جهت سنجش میزان اکراتوکسین، روش enzyme-linked immunosorbent assay (ELISA) استفاده گردید. داده‌ها با استفاده از آزمون‌های ANOVA و Tukey مورد تجزیه و تحلیل قرار گرفت. یافته‌ها: 70 درصد نمونه‌های مورد بررسی، آلودگی به اکراتوکسین A را نشان دادند. محدوده غلظت اکراتوکسین، صفر تا 61/5 نانوگرم در گرم و میانگین آلودگی نیز 19/1 ± 94/0 نانوگرم در گرم به دست آمد. آلودگی در بین برنج‌های داخلی، 2/1 نانوگرم در گرم و به ‌طور معنی‌داری بالاتر از میزان آلودگی در انواع برنج‌های خارجی بود (05/0 > P). غلظت اکراتوکسین تنها در یک نمونه بیش از حداکثر مجاز تعیین ‌شده توسط اتحادیه اروپا برای غلات (5 نانوگرم در گرم) گزارش گردید. یافته‌ها به ‌روشنی کاهش میزان اکراتوکسین A را در مرحله پخت نشان داد (05/0 > P) و نمونه‌های برنج پخته ‌شده به روش آبکش به نسبت پخت کته، آلودگی کمتری بر مبنای وزن خشک داشتند (05/0 > P). میزان کاهش در پخت آبکش و کته‌ای به ترتیب 21/17 و 24/14 درصد برآورد شد. نتیجه‌گیری: نتایج به دست آمده وضعیت آلودگی نمونه‌های برنج را برای سلامت عموم مخاطره‌آمیز نشان نمی‌دهد. فرایند پخت در کاهش این سم قارچی اثربخش می‌باشد و روش پخت آبکش در مقایسه با روش کته تأثیر بیشتری دارد.

Title: بررسی تأثیر کیفیت ارتباط پزشک- بیمار بر تبعیت دارویی بیماران مبتلا به پرفشاری خون مرکز قلب تهران

http://hsr.mui.ac.ir/index.php/jhsr/article/view/3294

مقدمه: پرفشاری خون، از مهم‌ترین عوامل خطر بروز بیماری‌های قلبی- عروقی می‌باشد. تحقیقات اخیر تأثیر عوامل گوناگون بر تبعیت دارویی را در این بیماری نشان داده‌اند. پژوهش حاضر با هدف تعیین تأثیر کیفیت ارتباط پزشک- بیمار بر میزان تبعیت دارویی مبتلایان به پرفشاری خون انجام شد. روش‌ها: در این مطالعه مقطعی، 62 بیمار مبتلا به پرفشاری خون از نوع اولیه که به مرکز قلب تهران مراجعه کرده بودند، به روش نمونه‌گیری در دسترس انتخاب شدند. جهت جمع‌آوری داده‌ها، از نسخه کوتاه پرسش‌نامه پیمان کاری (Working Alliance Inventory-Short Revised یا WAI-SR) و مقیاس خودکارامدی تبعیت دارویی (Medication Adherence Self-efficacy Scale یا MASES) استفاده گردید. در نهایت، داده‌ها با استفاده از مدل رگرسیون خطی چند متغیره در نرم‌افزار SPSS مورد تجزیه و تحلیل قرار گرفت. یافته‌ها: تأثیر معنی‌دار کیفیت ارتباط پزشک- بیمار بر تبعیت دارویی (002/0 = P، 996/5 = (58 و 3)F، 234/0 = 2R) با اندازه اثر متوسط و فاصله اطمینان 95 درصد، از 045/0 تا 402/0 به دست آمد. از میان مؤلفه‌های پیمان کاری نیز تنها مؤلفه تکالیف درمان تأثیر معنی‌داری بر مصرف داروها توسط بیمار داشت [621/9-787/0 = Confidence interval (CI) 95 درصد، 023/0 = P، 425/2 = (58)t]. نتیجه‌گیری: نتایج مطالعه حاضر شواهد ارزشمندی پیرامون نقش ارتباط درمانی در تبعیت دارویی فراهم نمود که این نتایج با توجه به ماهیت ارتباط درمانی مورد بحث قرار گرفته است.

Title: روان‌سنجی ابزار رفاه روانی، جسمی و معنوی و خرده مقیاس‌های آن در بیماران مبتلا به دیابت نوع دو

http://hsr.mui.ac.ir/index.php/jhsr/article/view/3249

مقدمه: سلامتي، مفهومي متأثر از مجموعه پيچيده‌اي از عوامل جسمی، رواني- اجتماعی، مذهبي، محيطي و فرهنگی است. هدف از انجام مطالعه حاضر، روان‌سنجی نسخه فارسی ابزار رفاه روانی، جسمی و معنوی (Mental, Physical and Spiritual Well-being Scale یا MPS) و خرده مقیاس‌های آن در بیماران مبتلا به دیابت نوع دو بود. روش‌ها: پس از بررسی ابزارهای موجود و با در نظر گرفتن شرایط فرهنگی و اجتماعی جامعه ایران، ابزار MPS تهیه شده توسط Vella-Brodrick و Allen انتخاب و ترجمه- بازترجمه شد. این پژوهش توصیفی- تحلیلی، بر روی 500 نفر از بیمارن مبتلا به دیابت نوع دو که به روش نمونه‌گیری خوشه‌ای چند مرحله‌ای انتخاب شده بودند، انجام گرفت. داده‌ها با استفاده از تحلیل عاملی اکتشافی در نرم‌افزار SPSS تجزیه و تحلیل گردید. آزمون‌های پایایی در دو بعد تکرارپذیری (شاخص همبستگی درونی خوشه‌ای) و سازگاری درونی (Cronbach¢s alpha) مورد استفاده قرار گرفت. از آنالیز عاملی تأییدی جهت تعیین تعداد شاخص‌های پرسش‌نامه و از آزمون‌های Kaiser-Meyer-Olkin (KMO) و کرویت Bartlett  به ترتیب برای تست پیش‌فرض‌های تحلیل عاملی تأییدی و کفایت نمونه استفاده شد. یافته‌ها: ضریب اعتبار ابزار بر پایه Cronbach¢s alpha، 58/0به دست آمد. 22 سؤال در سه حیطه تعیین شده، 71/42 درصد از کل واریانس را تبیین نمود. همبستگی بین متغیرهای پنهان و آشکار نیز بیشتر از 5/0 بود که شواهدی از اعتبار همگرا را نشان داد. نتیجه‌گیری: نتایج بررسی روان‌سنجی نسخه فارسی ابزار MPS مطابق با ابزار اصلي تهیه شده توسط Vella-Brodrick و Allen، در حد مرزی بود. پیشنهاد می‌شود مطالعات آینده با ایجاد تغییرات در شرایط دموگرافیک و یا تغییر در گروه هدف، دوباره مورد ارزیابی قرار گیرد.

Title: بررسی وضعیت کم‌خونی ناشی از فقر آهن در دانش‌آموزان دبستانی شهر اصفهان و تأثیر تغذیه بر بهبود آن

http://hsr.mui.ac.ir/index.php/jhsr/article/view/3266

مقدمه: مطالعه حاضر با هدف بررسي شيوع كم‌خوني ناشی از فقر آهن و تأثیر تغذیه مناسب دارای آهن بر رفع کمبود این عنصر معدنی در دانش‌آموزان دبستانی شهر اصفهان انجام شد. روش‌ها: در این پژوهش مداخله‌ای، 200 دانش‌آموز 7 تا 12 ساله دو دبستان شهر اصفهان به شیوه تصادفی انتخاب شدند و پس از اندازه‌گیری نمایه توده بدنی (Body mass index یا BMI)، نمونه‌ خون آن‌ها جهت بررسی شاخص‌های مورد نظر گرفته شد. سپس 35 نفر از دانش‌آموزان دارای فریتین کمتر از 12 میکروگرم در لیتر و هموگلوبین کمتر از 12 گرم در دسی‌لیتر انتخاب شدند و با ارایه آموزش و الگوی غذایی مناسب، پس از سه ماه مورد بررسی قرار گرفتند. داده‌ها در نرم‌افزار SPSS و سطح معنی‌داری 050/0 تجزیه و تحلیل گردید. یافته‌ها: شیوع کم‌خونی، فقر آهن و کم‌خونی ناشی از فقر آهن به ترتیب 0/6، 0/8 و 5/3 درصد بود. مقدار فریتین و هموگلوبین قبل از رژیم غذایی به ترتیب 11/10 ± 59/27 میکروگرم در لیتر و 97/0 ± 04/13 گرم در دسی‌لیتر گزارش شد که پس از مداخلات تغذیه‌ای افزایش یافت (به ترتیب 07/10 ± 88/30 میکروگرم در لیتر و 87/0 ± 35/13 گرم در دسی‌لیتر). طی سه ماه پژوهش، اختلاف معنی‌داری بین میانگین هموگلوبین و فریتین در سطح اطمینان 95 درصد مشاهده گردید. نتیجه‌گیری: تغذیه مناسب با مواد حاوی آهن، می‌تواند سبب بهبود آهن موجود در سرم خون کودکان مبتلا به کم‌خونی ناشی از فقر آهن شود. همچنین، پيشنهاد مي‌گردد برنامه غربالگری کودکان سن رشد در نظر گرفته شود و در صورت لزوم تحت درمان قرار گیرند.

Title: بررسی ارتباط عوامل زمینه‌ای با مصرف فست‌فود در دانش‌آموزان سال دوم دبیرستان شهر اصفهان

http://hsr.mui.ac.ir/index.php/jhsr/article/view/3246

مقدمه: مصرف فست‌فود طی دهه‌های گذشته در میان نوجوانان افزایش چشمگیری یافته است. با توجه به این که بسیاری از عادات از جمله عادات غذایی نامناسب، در سنین نوجوانی شکل می‌گیرد و در سال‌های بعدی عمر باقی می‌ماند و زمینه‌ساز بسیاری از بیماری‌های مزمن می‌شود، مطالعه حاضر با هدف بررسی ارتباط برخی عوامل زمینه‌ای با مصرف فست‌فود در دانش‌آموزان شهر اصفهان انجام شد. روش‌ها: این پژوهش از نوع توصیفی- تحلیلی و جامعه هدف آن متشکل از 400 دانش‌آموز 16 ساله ساکن شهر اصفهان در سال تحصیلی 95-1394 بود. نمونه‌ها متناسب با حجم و بر حسب جنسیت، از نواحی شش‌گانه آموزش و پرورش شهر اصفهان انتخاب شدند. ابزار جمع‌آوری اطلاعات، پرسش‌نامه‌ای محقق ساخته شامل دو بخش اطلاعات دموگرافیک و رفتار تغذیه‌ای بود. داده‌ها با استفاده از آزمون‌های Independent t و آزمون همبستگی Spearman در نرم‌افزار SPSS مورد تجزیه و تحلیل قرار گرفت. یافته‌ها: شرکت‌کنندگان مطالعه، 4۰۰ نفر از دانش‌آموزان سال دوم دبیرستان (پایه یازدهم) و 200 دختر (۵۰ درصد) و ۲۰۰ پسر (۵۰ درصد) بودند. تفاوت معنی‌داری بین میانگین نمره مصرف فست‌فود در دانش‌آموزان دختر (58/0 ± 35/9) و پسر (71/0 ± 16/11) وجود نداشت. (080/0 = P). بر اساس نتایج آزمون Spearman، بین میانگین نمره مصرف فست‌فود با تحصیلات پدر و مادر و درامد خانواده رابطه معنی‌دار و مستقیمی مشاهده شد (001/0 > P)، اما با امکانات و تسهیلات زندگی ارتباط معنی‌داری را نشان نداد. نتیجه‌گیری: انجام مداخلات در جهت اتخاذ رفتارهای تغذیه‌ای سالم و کاهش مصرف فست‌فود در مدارس باید با در نظر گرفتن وضعیت اقتصادی- اجتماعی دانش‌آموزان و تحصیلات والدین آن‌ها صورت پذیرد تا بتواند در درازمدت نقش مهمی در اتخاذ سبک زندگی سالم داشته باشد.

Title: بررسی سطح سواد سلامت زنان شهرستان بردسکن در سال 1395: یک مطالعه مقطعی

http://hsr.mui.ac.ir/index.php/jhsr/article/view/3302

مقدمه: سواد سلامت، توانایی افراد در به دست آوردن، تحلیل کردن و درک اطلاعات و خدمات اولیه بهداشتی مورد نیاز جهت تصمیم‌گیری در مورد مسایل سلامتی و بهداشتی می‌باشد. مطالعه حاضر با هدف بررسی سطح سواد سلامت زنان شهرستان بردسکن و عوامل مؤثر بر آن طراحی و اجرا گردید. روش‌ها: این پژوهش از نوع توصیفی- تحلیلی- مقطعی بود که در سال 1395، بر روی 431 نفر از زنان 18 تا 65 ساله ساکن شهرستان بردسکن انجام گرفت. نمونه‌ها با استفاده از روش نمونه‌گیری خوشه‌ای انتخاب شدند. داده‌ها به وسیله پرسش‌نامه سواد سلامت ایرانیان (Health Literacy for Iranian Adults یا HELIA) جمع‌آوری شد و سپس با استفاده از آزمون‌های Kolmogorov-Smirnov، Independent t ، Mann–Whitney و 2c در نرم‌افزار SPSS مورد تجزیه و تحلیل قرار گرفت. یافته‌ها: میانگین سنی شرکت‌کنندگان، 74/8 ± 90/29 سال بود. ارتباط معنی‌داری بین سطح سواد سلامت با سن، تأهل، تحصیلات، سابقه بیماری و شغل مشاهده شد. نمره سواد سلامت در نمونه‌ها، 16/16 ± 60/67 از 100 و در حد متوسط گزارش گردید. مهم‌ترین منابع کسب اطلاعات مربوط به سلامت از دیدگاه شرکت‌کنندگان به ترتیب مربوط به پزشکان و کارکنان بهداشتی، اینترنت و رادیو و تلویزیون بود. 57 درصد از افرادی که سابقه بیماری گزارش کرده بودند، سواد سلامت کافی نداشتند. نتیجه‌گیری: به طور کلی، سواد سلامت بیشتر زنان مطالعه حاضر در حد متوسطی بود. با توجه به نقش مهم زنان در تربیت خانواده و جامعه و تأثیر سواد سلامت بر این نقش مهم، ﻻﺯﻡ ﺍﺳﺖ ﺑﺮﻧﺎﻣﻪ‌ﻫﺎﻯ ﺁﻣﻮﺯﺷﻰ ﺑﺎ ﻫﺪﻑ ﺍﺭﺗﻘﺎﻯ سطح سواد سلامت زنان و به دنبال آن، مهارت‌های خودمراقبتی آنان طراحی و اجرا گردد.

Title: تأثیر مداخله آموزشی مبتنی بر تئوری رفتار برنامه‌ریزی شده بر میزان فعالیت فیزیکی و تبعیت دارویی در بیماران مبتلا به دیابت نوع دو

http://hsr.mui.ac.ir/index.php/jhsr/article/view/3201

مقدمه: خودمراقبتی توسط بیماران مبتلا به دیابت، نقشی حیاتی در کنترل این بیماری و پیشگیری از عوارض ناشی از آن ایفا می‌کند. انجام فعالیت‌های فیزیکی و پایبندی به درمان دارویی، از جمله رفتارهای خودمراقبتی در بیماران مبتلا به دیابت است. مطالعه حاضر با هدف بررسی تأثیر مداخله آموزشی مبتنی بر تئوری رفتار برنامه‌ریزی شده بر میزان فعالیت فیزیکی و تبعیت دارویی در بیماران مبتلا به دیابت نوع دو انجام شد. روش‌ها: این پژوهش شبه تجربی از نوع کارآزمایی تصادفی شده با گروه شاهد بود که در سال 1395 بر روی 160 نفر از بیماران مبتلا به دیابت نوع دو مراجعه‌کننده به مراکز بهداشتی- درمانی شهر یاسوج انجام گردید. نمونه‌ها با استفاده از روش نمونه‌گیری خوشه‌ای تصادفی انتخاب شد. داده‌های به دست آمده از هر پرسش‌نامه در نرم‌افزار SPSS مورد تجزیه و تحلیل قرار گرفت. یافته‌ها: بعد از مداخله آموزشی، تفاوت معنی‌داری بین همه سازه‌ها به جز سازه هنجارهای انتزاعی مربوط به تبعیت دارویی مشاهده شد. همچنین، متغیرهای فعالیت فیزیکی و تبعیت دارویی بعد از مداخله اختلاف معنی‌داری را نشان دادند. نتیجه‌گیری: انجام مداخلات آموزشی بر مبنای تئوری رفتار برنامه‌ریزی شده، تأثیر مثبت و معنی‌داری بر میزان فعالیت فیزیکی و تبعیت دارویی بیماران مبتلا به دیابت نوع دو دارد.

Title: بررسی وضعیت مدیریت پسماندهای عفونی در بیمارستان‌های استان بوشهر طی سال‌های 95-1394

http://hsr.mui.ac.ir/index.php/jhsr/article/view/3050

مقدمه: هدف از انجام مطالعه حاضر، تعیین وضعیت پسماندهای عفونی و بررسی مدیریت آن در بیمارستان‌های استان بوشهر طی سال‌های 95-1394 بود. روش‌ها: این پژوهش از نوع توصیفی- مقطعی بود که با تکمیل پرسش‌نامه، مصاحبه، مشاهده، ممیزی، توزین و تست‌های بی‌خطرسازی طی مدت 24 ماه در 10 بیمارستان استان بوشهر انجام شد. پرسش‌نامه نهایی از 103 سؤال تشکیل شد و بر اساس رهنمودهای سازمان جهانی بهداشت، برنامه مدیریت پسماندهای پزشکی و رهنمودهای تخصصی مرکز سلامت محیط و کار به اجرا درآمد. یافته‌ها: در 10 بیمارستان مورد بررسی، روزانه 34/5718 کیلوگرم پسماند در بیمارستان‌های استان بوشهر تولید می‌گردد که شامل پسماند عادی (61/59 درصد) و خطرناک (39/40 درصد) می‌باشد. در هیچ یک از بیمارستان‌ها بازیافت پسماندهای عفونی و در 2 بیمارستان نیز عملیات بی‌خطرسازی انجام نمی‌شد؛ در حالی که برنامه‌های تفکیک این پسماندها در همه بیمارستان‌ها صورت می‌گرفت. همه کارکنان خدماتی در هنگام جابه‌جایی پسماند، از وسایل حفاظت فردی استفاده می‌کردند. تمامی بیمارستان‌ها جایگاه ویژه پسماند داشتند و تنها 6 بیمارستان دارای جایگاه ویژه پسماند خطرناک بود و حمل پسماندها از بیمارستان تا محل دفع نهایی در 5 بیمارستان مطلوب عنوان شد. نتیجه‌گیری: با وجود تلاش فراوان در مدیریت پسماندهای پزشکی، هنوز مشکلاتی در فرایند مدیریت به ویژه تفکیک، حمل و نقل، نگهداشت موقت و بی‌خطرسازی وجود دارد که نیازمند آموزش مداوم رده‌های پرسنلی، نظارت دقیق و الزام‌آور بر سیستم بی‌خطرسازی، امحا و دفن نهایی در فیلد با در نظر گرفتن شاخص‌های زیست محیطی و مهندسی می‌باشد.

Title: نقش عوامل کلان اقتصادی در فضای کسب و کار بنگاه‌های کوچک و متوسط بخش سلامت استان اصفهان بر اساس مدل PESTEL (مطالعه موردی: صنعت دارو)

http://hsr.mui.ac.ir/index.php/jhsr/article/view/3255

مقدمه: مطالعه حاضر با تمرکز بر نقش عوامل برون بنگاهی، به بررسی نقش عوامل کلان در فضای کسب و کار بنگاه‌های کوچک و متوسط صنعت داروی استان اصفهان پرداخت. روش‌ها: این پژوهش از نظر هدف، کاربردی بود و به روش توصیفی- پیمایشی انجام شد. داده‌ها به روش میدانی و به وسیله پرسش‌نامه محقق ساخته مبتنی بر مدل PESTEL (Political, Economic, Social, Technological, Environmental, Legal) جمع‌آوری گردید. جامعه آماری مطالعه متشکل از 238 نفر از فعالان صنعت داروی استان بود که از این تعداد بر اساس جدول Krejcie و Morgan، 142 نفر به روش طبقه‌ای در دسترس انتخاب شدند. روایی صوری و محتوایی پرسش‌نامه مورد بررسی و تأیید قرار گرفت و پایایی آن نیز با استفاده از ضریب Cronbach¢s alpha، 95/0 به دست آمد. جهت بررسی نقش عوامل شش‌گانه موجود در الگوی PESTEL (عوامل سیاسی، اقتصادی، اجتماعی، تکنولوژی، محیط زیست و قانونی) از آزمون‌های t تک متغیره و رتبه‌بندی Friedman استفاده شد. یافته‌ها: همه عوامل شش‌گانه مدل PESTEL تأثیر معنی‌داری بر فضای کسب و کار بنگاه‌های کوچک و متوسط صنعت داروی استان اصفهان داشت. در این میان، محیط سیاسی در رتبه نخست تأثیرگذاری قرار گرفت و آخرین رتبه نیز به محیط تکنولوژی اختصاص یافت. نتیجه‌گیری: عواملی همچون تغییرات سیاسی، سیاست‌های دولت در بازار دارو، ثبات نرخ ارز، ثبات قوانین و مقررات، طرح تحول نظام سلامت، مسایل زیست ‌محیطی، الگو و فرهنگ مصرف دارو، قوانین تولید و توزیع دارو و دیگر تغییرات محیطی مشابه بر فضای کسب و کار در صنعت دارو تأثیر می‌گذارد.

Title: ارزیابی جلیقه خنک‌کننده ایرانی (ژل یخ اسپادانا) و جلیقه خنک‌کننده تغییر فاز (پارافینی) بر روی شاخص‌های استرین فیزیولوژیکی کارگران در شرایط گرم و مرطوب عسلویه

http://hsr.mui.ac.ir/index.php/jhsr/article/view/3009

مقدمه: جهت پیشگیری از استرس گرمایی در محیط‌های کاری گرم، کاهش سطح استرس حرارتی کارگران امری ضروری است. هدف از انجام مطالعه حاضر، بررسی و مقایسه تأثیر دو نوع جلیقه خنک‌کننده [جلیقه خنک‌کننده ایرانی (ژل یخ اسپادانا) و جلیقه خنک‌کننده تغییر فاز (پارافینی)] بر روی شاخص استرین فیزیولوژیکی (Physiological strain index یا PSI) کارگران در شرایط گرم و مرطوب عسلویه بود. روش‌ها: این پژوهش به روش مقطعی و مداخله‌ای بر روی 90 نفر از کارگران با سطح فعالیت جسمانی و لباس یکسان در منطقه عسلویه با میانگین دمایی 47 درجه سانتی‌گراد و رطوبت نسبی 95 درصد در تابستان 1395 انجام شد. ضربان قلب، دمای دهانی، میزان تعریق و شاخص Wet-bulb globe temperature (WBGT) به مدت 120 دقیقه در هنگام استفاده از دو نوع جلیقه‌ها و در گروه شاهد اندازه‌گیری گردید. یافته‌ها: میانگین شاخص WBGT برای هر سه گروه 65/37 درجه سانتی‌گراد به دست آمد. میانگین ضربان قلب در گروه استفاده‌کننده از جلیقه خنک‌کننده ایرانی و پارافینی و شاهد به ترتیب 76/53، 91/89 و 07/97 ضربان در دقیقه، میانگین دمای دهانی به ترتیب 70/36، 63/78 و 36/36 درجه سانتی‌گراد و میانگین PSI به ترتیب 18/3، 29/2 و 12/6 بود. اختلاف معنی‌داری در میزان PSI بین گروه‌های مورد و گروه شاهد وجود داشت (001/0 > P). نتیجه‌گیری: جلیقه خنک‌کننده پارافینی و جلیقه خنک‌کننده ایرانی ژل یخ اسپادانا می‌توانند در شرایط گرم و مرطوب عسلویه در فصل تابستان به مدت90 دقیقه سطح استرس حرارتی کاربران را تا میزان قابل قبول و مناسبی کاهش دهند.

Title: بررسی تأثیر سموم ارگانوفسفره بر تغییرات سطح فعالیت آنزیم کولین استراز پلاسمای کشاورزان

http://hsr.mui.ac.ir/index.php/jhsr/article/view/3186

مقدمه: ترکیبات ارگانوفسفره، دسته‌ای از آفت‌کش‌ها با کاربردهای شناخته شده در کشاورزی هستند که مسمومیت با آن‌ها، مشکلات بزرگی را در سراسر جهان به ‌ویژه در کشورهای در حال ‌توسعه ایجاد کرده است. مطالعه حاضر با هدف بررسی تأثیر سموم ارگانوفسفره بر فعالیت آنزیم کولین استراز پلاسمای کشاورزان صورت گرفت. روش‌ها: این پژوهش به روش توصیفی- تحلیلی انجام شد. گروه هدف، 17 نفر از کشاورزان مرد سم‌پاش بودند که با سموم ارگانوفسفره مواجهه داشتند. 17 نفر نیز از میان افراد غیر کشاورز به ‌عنوان گروه شاهد مورد بررسی قرار گرفتند. قبل و پس از اجرای عملیات سم‌پاشی، یک نمونه خون از گروه کشاورزان و گروه شاهد گرفته شد و فعالیت آنزیم کولین استراز پلاسمای آن‌ها با دستگاه فتومتریک بررسی گردید. یافته‌ها: میانگین سطح فعالیت آنزیم کولین استراز پلاسما قبل از سم‌پاشی در گروه مورد (کشاورزان مواجهه یافته با سموم ارگانوفسفره)، 2/12086 میلی‌گرم بر لیتر بود که این میزان پس از سم‌پاشی به 3/8516 میلی‌گرم بر لیتر کاهش یافت. میانگین سطح فعالیت این آنزیم در گروه شاهد نیز 8/13655میلی‌گرم بر لیتر گزارش شد. نتیجه‌گیری: با توجه به اهميت ارزيابي تماس کشاورزان با سموم ارگانوفسفره و تأثیرگذاری بر سطح آنزیم کولین استراز، می‌توان گفت که آموزش کشاورزان در جهت تولید محصولات ارگانیک به‌ منظور استفاده هرچه کمتر از سموم و جلوگیری از مسمومیت‌های ناشی از سم‌پاشی در این قشر ضروری به نظر می‌رسد.

Title: کمی‌سازی اثرات بهداشتی PM10 هوای شهرستان گچساران با استفاده از نرم‌افزار AirQ در سال 1394

http://hsr.mui.ac.ir/index.php/jhsr/article/view/3127

مقدمه: ذرات معلق هوا برای سلامتی انسان بسیار خطرناک می‌باشد. تماس طولانی مدت با این ذرات معلق، شاخص‌ مهمی در میزان مرگ و میر بر اثر سرطان ریه و بیماری‌های قلبی به شمار می‌رود. هدف از انجام مطالعه حاضر، برآورد اثرات بهداشتی منتسب به آلاینده ذرات معلق کمتر از 10 میکرون (Particulate matter یا PM10) هوای شهرستان گچساران بود. روش‌ها: داده‌های مورد نیاز این پژوهش توصیفی، از اداره کل محیط زیست استان کهگیلویه و بویراحمد اخذ شد و در محیط Excel مورد پردازش قرار گرفت. سپس جهت برآورد خطر نسبی (Relative risk یا RR) و جزء منتسب (Attributable portion یا AP)، داده‌ها در نرم‌افزار AirQ تجزیه و تحلیل گردید. یافته‌ها: متوسط غلظت سالیانه PM10 در شهرستان گچساران در سال 1394، حدود 121 میکروگرم بر مترمکعب به دست آمد. از کل 58 مورد مرگ منتسب به PM10، 27 مورد ناشی از بیماری‌های قلبی- عروقی و 13 مورد ناشی از بیماری‌های تنفسی بود. همچنین، تعداد تجمعی موارد بستری در بیمارستان به دلیل بیماری‌های قلبی- عروقی و تنفسی به ترتیب 39 و 147 نفر گزارش گردید. نتیجه‌گیری: آلودگی هوای ناشی از PM10 در شهر گچساران، باعث تعداد زیادی مرگ و بستری شدن در سال 1394 شده است. بنابراین، انتظار می‌رود که مسؤولان از برنامه‌های کوتاه مدت، میان مدت و بلند مدت جهت کنترل این نوع از آلاینده‌ها استفاده نمایند.

Title: تأثير هشت هفته تمرینات منتخب اصلاحی بر روی زاویه کیفوز پسران نوجوان مبتلا به کیفوزیس

http://hsr.mui.ac.ir/index.php/jhsr/article/view/3159

مقدمه: کیفوزیس، افزایش غیر طبیعی قوس ناحیه پشتی می‌باشد که با کشیده شدن اکستنسورهای فقرات و ضعیف شدن عضله ذوزنقه‌ای میانی و تحتانی و کوتاهی ابداکشن عضلات سینه‌ای بزرگ و کوچک و بین دنده‌ای همراه است. بنابراین، ضرورت و اهمیت انجام چنین تحقیقاتی به خصوص در بین افراد کم سن و سال احساس می‌گردد؛ چرا که به علت انعطاف استخوان‌ها و رشد اندام‌های بدن در این سنین، می‌توان با استفاده از حرکات اصلاحی مناسب، در رفع ناهنجاری‌های آن‌ها اقدام نمود و راهکارهای مناسب را در اختیار آنان قرار داد. هدف از انجام پژوهش حاضر، تأثير 8 هفته تمرینات منتخب اصلاحی بر روی زاویه کیفوز پسران نوجوان مبتلا به کیفوزیس بود. روش‌ها: 24 نفر از پسران باشگاهی 15 تا 18 ساله در اصفهان به عنوان نمونه آماري انتخاب شدند. برای اندازه‌گیری زاویه کیفوز، از خط‌کش منعطف استفاده گردید. تمرینات کششی، مقاومتی و خودتحرک‌بخشی به مدت 8 هفته انجام گرفت. در نهایت، داده‌ها با استفاده از آزمون‌های Paired t و تحلیل كوواريانس مورد تجزيه و تحليل قرار گرفت. يافته‌ها: ميزان زاویه کیفوز در نوجوانان پسر مبتلا به کیفوزیس در پیش‌آزمون 9/0 ± 8/48 درجه بود (604/0 = P، 275/0 = F) که پس از استفاده از تمرينات منتخب اصلاحی، كاهش معني‌داري را نشان داد (8/0 ± 9/46 درجه) (001/0 = P، 068/1680 = F). نتیجه‌گیری: بر اساس نتایج به دست آمده، تمرینات اصلاحی به تنهایی و به مدت 3 روز در هفته و طی 8 هفته متوالی، تأثیر مثبتی بر بهبود زاویه کیفوز آزمودنی‌ها دارد.

Title: میزان و علل مرگ و میر پری ناتال در شهرستان کرمانشاه (93-1390)

http://hsr.mui.ac.ir/index.php/jhsr/article/view/3147

مقدمه: شاخص مرگ پری ناتال یک شاخص مهم بهداشتی و ارزیابی سلامت است که تأثیر مستقیم بر شاخص مرگ و میر شیرخواران دارد. هدف از انجام مطالعه حاضر، تعیین میزان و علل مرگ و میر پری ناتال در شهرستان کرمانشاه بود. روش‌ها: در مطالعه مقطعی حاضر موارد ثبت شده مرگ و میر پری ناتال شهر کرمانشاه طی سال‏های 93-1390 استخراج شد. اطلاعات شامل مشخصات نوزاد، اطلاعات مربوط به مادر، شاخص‌هاي بارداري و زایمان و علت اصلی مرگ بر اساس International Classification of Diseases 10th (ICD-10) جمع‌آوری شد. اطلاعات با استفاده از میانگین و انحراف معیار برای متغیرهای کمی، تعداد و درصد برای متغیرهای کیفی و نرم‌افزار Stata تجزیه و تحلیل شد. یافته‌ها: از 1236 مرگ پری ناتال، 1/56 درصد پسر بودند. بیشترین مرگ و میر در گروه وزنی، کم و بسیار کم و شایع‌ترین بیماری زمینه‌ای مادر، پرفشاری خون بود. بیشترین میزان مرگ پری ناتال مربوط به سال 91 (3/12 مرگ در هر هزار تولد) و کمترین میزان مربوط به سال 93 (7/5 مرگ در هر هزار تولد) بود. شایع‌ترین علت مرگ پری ناتال با 6/87 درصد مربوط به گروه اختلالات مشخص با منشأ دوران پیرامون تولد بود. نتیجه‌گیری: با توجه به نتایج، تأکید بر مراقبت‏های کافی در دوران حاملگی و پری ناتال، شناسایی و پیگیری دقیق‌تر مادران پرخطر و دارای بیماری‏های زمینه‏ای، پیشگیری از زایمان زودرس و تولد نوزادان دارای کم‏وزنی شدید می‌تواند در کاهش مرگ و میرهای پری ناتال مؤثر باشد.

Title: تعیین ضرایب سینتیکی در تصفيه فاضلاب شهری به روش لاگون هوادهي در مقیاس کامل (مطالعه موردي: تصفيه‌خانه فاضلاب سپاهان‌شهر اصفهان)

http://hsr.mui.ac.ir/index.php/jhsr/article/view/3035

مقدمه: در گذشته طراحی فرایندهای تصفیه فاضلاب بر اساس شاخص‌های تجربی انجام می‌گرفت، اما به دلیل کمیت و کیفیت متغیر فاضلاب ورودی به تصفیه‌خانه، استفاده صرف از شاخص‌های استاندارد نمی‌تواند کیفیت پساب خروجی از تصفیه‌خانه را تضمین نماید. امروزه علاوه بر شاخص‌های تجربی، عوامل منطقی بر اساس معادلات سینتیکی بیولوژیکی نیز در طراحی استفاده می‌شوند. هدف از انجام مطالعه حاضر، تعیین ضرایب بیوسینتیکی KB و Umax با استفاده از مدل Kincannon-Stover در لاگون هوادهی تصفیه‌خانه فاضلاب سپاهان‌شهر به منظور استفاده از این شاخص‌ها برای طراحی سیستم‌هایی در شرایط مشابه در کشور بود. روش‌ها: در این پژوهش، راندمان حذف Biochemical oxygen demand (BOD)، Chemical oxygen demand (COD) و کل جامدات معلق (Total suspended solids یا TSS)، طی یک دوره سه ساله در لاگون هوادهی تصفیه‌خانه فاضلاب سپاهان‌شهر بررسی و ضرایب بیوسینتیکی محاسبه گردید. نمونه‌برداری از ورودی و خروجی تصفیه‌خانه به صورت هفتگی انجام گرفت. یافته‌ها: میانگین راندمان حذف BOD، COD و TSS در لاگون هوادهی به ترتیب 72 ± 4، 70 ± 6 و 62 ± 6 درصد بود. ضرایب بیوسینتیکی با استفاده از مدل Kincannon-Stover محاسبه شد و مقادیر Umax و KB برای لاگون هوادهی به ترتیب 8/55 و 0/136 گرم COD بر لیتر در روز به دست آمد. نتیجه‌گیری: کیفیت پساب خروجی از تصفیه‌خانه فاضلاب سپاهان‌شهر مطابق با استانداردهای مورد نظر محیط زیست برای استفاده در کشاورزی است. مدل اصلاح شده Kincannon-Stover همبستگی بالایی (88/0 = 2R) با نتایج آزمایشگاهی دارد. بنابراین، می‌توان از این مدل جهت طراحی و پیش‌بینی رفتار لاگون‌های هوادهی استفاده نمود.

Title: بررسی تأثیر بار سطحی و زمان ماند بر میزان کارایی فرایند نیزار مصنوعی با جریان زیرسطحی کاشته شده با نی Phragmites australis در تصفیه تکمیلی پساب خروجی از تصفیه ثانویه صنایع دامپروری

http://hsr.mui.ac.ir/index.php/jhsr/article/view/3190

مقدمه: فاضلاب‌ تولید شده در اثر فعالیت‌های دامداری، مملو از مواد مغذی می‌باشد که در صورت ورود به آب‌های سطحی و زیرزمینی، اختلالات قابل توجهی را در کیفیت این منابع ایجاد خواهد کرد. نیزارهای مصنوعی به دلیل داشتن هزینه‌های به نسبت پایین احداث و بهره‌برداری نسبت به سیستم‌های تصفیه متداول و اثراتی که در حذف آلاینده‌ها دارد، یکی از گزینه‌های پیشنهادی جهت تصفیه فاضلاب‌های دامداری‌ها می‌باشد. هدف از انجام مطالعه حاضر، تعیین کارایی نیزارهای مصنوعی در حذف مواد آلی و نوترینت‌ها از پساب دامپروری و بررسی تأثیر بار سطحی و زمان ماند در کارایی نیزارهای مصنوعی بود. روش‌ها: در این پژوهش، راندمان حذف شاخص‌های Biochemical oxygen demand (BOD)، Chemical oxygen demand (COD)، کل جامدات معلق (Total suspended solids یا TSS)، نیترات- نیتروژن و فسفات (ارتوفسفات) از پساب دامپروری با استفاده از نیزارهای مصنوعی در چند زمان ماند و بارگذاری هیدرولیکی متفاوت طی یک دوره شش ماهه مورد بررسی قرار گرفت. یافته‌ها: مقایسه راندمان حذف نشان داد که بیشترین میانگین میزان حذف در نیزارهای مصنوعی، در زمان ماند بیشتر، بارگذاری هیدرولیکی کمتر و درجه حرارت بیشتر از 15 درجه سانتی‌گراد اتفاق افتاد و میزان حذف شاخص‌های BOD، COD، TSS، نیترات و فسفات به ترتیب 74، 62، 69، 61 و 23 درصد گزارش گردید. نتیجه‌گیری: کارایی نیزارهای مصنوعی در بهبود کیفیت پساب خروجی از تصفیه ثانویه دامداری‌ها به منظور دستیابی به استاندارد سازمان محیط زیست ایران جهت آبیاری فضای سبز قابل قبول می‌باشد.

Title: بررسی عملکرد ماسک‌های تنفسی مورد استفاده شاغلان نسوزکاری در کنترل مواجهه با آلاینده‌های ذره‌ای در یک صنعت تولید فولاد

http://hsr.mui.ac.ir/index.php/jhsr/article/view/3220

مقدمه: یکی از عوامل زیان‌آور در بعضی از محیط‌های کاری، گرد و غبار است. استفاده از ماسک‌های تصفیه کننده هوا نمونه‌ای از راه‌های کاهش مواجهه در محیط پر گرد و غبار می‌باشد. بنابراین، هدف از انجام مطالعه حاضر، بررسی عملکرد ماسک‌های تنفسی مورد استفاده در شاغلان نسوزکاری در کنترل مواجهه با آلاینده‌های ذره‌ای بود. روش‌ها: در این مطالعه عملکرد سه نوع ماسک در برابر آلاینده‌های ذره‌ای مورد ارزیابی قرار گرفت. جهت بررسی عملکرد، در حین کار فرد و به‌ طور همزمان غلظت آلاینده در هوای خارج ماسک و هوای داخل ماسک اندازه‌گیری شد و سپس، درصد کارایی و درصد نفوذ ماسک محاسبه گردید. یافته‌ها: میانگین درصد نفوذ در ماسک FFP3 بیشتر از دو نوع FFP2 و نیم صورت الاستومری با فیلتر P100 برآورد گردید. بررسی کارایی دو نوع ماسک FFP3 و FFP2 برحسب طول زمان مصرف نشان داد که طی چند ساعت اول مصرف، کارایی کاهش و سپس افزایش یافت. نتیجه‌گیری: با افزایش مقاومت سطح فیلتر میزان عبور جریان هوا از فضای بین ماسک و صورت افزایش می‌یابد که در نتیجه کارایی ماسک کم می‌شود. بنابراین، مقاومت سطح ماسک و نفوذ هوا از فضای خالی بین صورت فرد و ماسک فاکتور اصلی در تعیین کارایی ماسک می‌باشد.

Title: اندازه‌گیری غلظت هیدروکربن‌های آروماتیک چند حلقه‌ای در هوای آزاد مناطق پرترافیک اصفهان

http://hsr.mui.ac.ir/index.php/jhsr/article/view/3130

مقدمه: هیدروکربن‌های آروماتیک چند حلقه‌ای (Polycyclic Aromatic Hydrocarbons یا PAHs)، ترکیباتی هستند که به طور عمده از احتراق ناقص سوخت‌های فسیلی حاصل می‌شوند. این ترکیبات به دلیل پایداری، تجمع زیستی و اثرات سرطان‌زایی و جهش‌زایی، یکی از نگرانی‌های جوامع به شمار می‌روند. در مطالعه حاضر، غلظت PAHs در هوای تنفسی مناطق پرترافیک اصفهان در فصول تابستان و پاییز سال 1395 بررسی گردید. روش‌ها: نمونه‌برداری از هوا به روش منفعل و با استفاده از فیبرهای جاذب سرنگی Solid Phase Microextraction (SPME) انجام گرفت. جاذب‌ها در محفظه‌های نمونه‌برداری در مسیرهای پرتردد به مدت 24 ساعت نصب شد و پس از نمونه‌برداری، PAHs جذب شده در فاز جامد به طور مستقیم با دستگاه کروماتوگرافی گازی متصل به دتکتور اسپکترومتر جرمی سه‌گانه (Gas Chromatography/Triple quadrupole-mass spectrometer یا GC/TQ-MS) طبق روش‌های استاندارد تجزیه و تحلیل گردید. یافته‌ها: حداکثر میانگین ΣPAHs به میدان آزادی با غلظت 06/4 ± 83/53 نانوگرم در مترمکعب در فصل پاییز و حداقل میانگین نیز به پل وحید با غلظت 28/1 ± 82/21 نانوگرم در مترمکعب در فصل تابستان اختصاص داشت. غلظت Acenaphthylene بیش از سایر PAH‌ها شناسایی شد. میانگین غلظت PAHs در فصل پاییز بیشتر از فصل تابستان به دست آمد. نتیجه‌گیری: مواجهه با PAHs از طریق هوا در تمام ایستگاه‌ها به ویژه در مکان‌های پرترافیک، بالاتر از حدود اعلام شده توسط Occupational Safety and Health Administration (OSHA)، National Institute for Occupational Safety and Health (NIOSH) و American Conference of Governmental Industrial Hygienists (ACGIH) است که می‌تواند ناشی از ترافیک سنگین و تردد بیش از حد خودروها و همچنین، شرایط توپوگرافی ویژه شهر باشد. بنابراین، کنترل ترافیک و کنترل انتشار آلاینده‌ها از خودروها، تأثیر زیادی در کاهش میزان PAHs دارد.

Title: بررسی اثربخشی مداخلات مبتنی بر آسیب‌های برآمده از فضای مجازی بر کیفیت رابطه زوجین

http://hsr.mui.ac.ir/index.php/jhsr/article/view/3260

مقدمه: ماهیت رو به گسترش فضای مجازی و نقش بسزایی که در ارتباطات زوجین و کیفیت روابط زناشویی آن‌ها دارد، لزوم تدوین بسته مداخلاتی مؤثر برای کمک به زوج‌هایی که از فضای مجازی آسیب دیده‌اند و کیفیت رابطه آنان تحت تأثیر قرار گرفته است را ضروری می‌نماید. مطالعه حاضر با هدف اثربخشی مداخلات مبتنی بر آسیب‌های برآمده از فضای مجازی بر کیفیت رابطه زناشویی زوجین انجام گردید. روش‌ها: این پژوهش به روش نیمه تجربی انجام شد و نمونه‌ها از بین زوجین آسیب دیده از فضای مجازی که به فرهنگسراها مراجعه نموده و پرسش‌نامه محقق ساخته آسیب‌های برآمده از فضای مجازی را تکمیل کرده بودند و نمره آسیب‌های آن‌ها به طور معنی‌داری بالاتر از میانگین بود، انتخاب شدند و به صورت تصادفی در دو گروه آزمایش و شاهد قرار گرفتند. مداخله طی 10 جلسه آموزشی بر روی گروه آزمایش اجرا شد. داده‌ها با کمک پرسش‌نامه‌ آسیب‌های برآمده از فضای مجازی در رابطه زوجین و پرسش‌نامه کیفیت رابطه زناشویی (Perceived Relationship Quality Components یا PRQC) جمع‌آوری گردید و در نهایت، با استفاده از آزمون تحلیل کواریانس چند متغیره در نرم‌افزار SPSS مورد تجزیه و تحلیل قرار گرفت. یافته‌ها: آموزش درمان مبتنی بر آسیب‌های برآمده از فضای مجازی، تأثیر معنی‌داری بر کیفیت رابطه زناشویی زوجین گروه آزمایش داشت؛ بدین معنی که میانگین کیفیت رابطه زناشویی گروه آزمایش در مرحله پس‌آزمون به طور معنی‌داری بالاتر از گروه شاهد بود (050/0 > P). نتیجه‌گیری: برنامه مشاوره‌ای تدوین شده به روش زوجی می‌تواند به عنوان روش مؤثری در ارتقای کیفیت زندگی زوجین آسیب دیده از فضای مجازی مورد استفاده قرار گیرد.
